# Early Transcriptional Signatures of the Immune Response to a Live Attenuated Tetravalent Dengue Vaccine Candidate in Non-human Primates

**DOI:** 10.1371/journal.pntd.0004731

**Published:** 2016-05-23

**Authors:** Fiona R. Strouts, Stephen J. Popper, Charalambos D. Partidos, Dan T. Stinchcomb, Jorge E. Osorio, David A. Relman

**Affiliations:** 1 Department of Microbiology and Immunology, Stanford University, Stanford, California, United States of America; 2 Takeda Vaccines, Inc., Deerfield, Illinois, United States of America; 3 Department of Medicine, Stanford University, Stanford, California, United States of America; 4 Veterans Affairs Palo Alto Health Care System, Palo Alto, California, United States of America; University of Rhode Island, UNITED STATES

## Abstract

**Background:**

The development of a vaccine against dengue faces unique challenges, including the complexity of the immune responses to the four antigenically distinct serotypes. Genome-wide transcriptional profiling provides insight into the pathways and molecular features that underlie responses to immune system stimulation, and may facilitate predictions of immune protection.

**Methodology/Principal Findings:**

In this study, we measured early transcriptional responses in the peripheral blood of cynomolgus macaques following vaccination with a live, attenuated tetravalent dengue vaccine candidate, TDV, which is based on a DENV-2 backbone. Different doses and routes of vaccine administration were used, and viral load and neutralizing antibody titers were measured at different time-points following vaccination. All 30 vaccinated animals developed a neutralizing antibody response to each of the four dengue serotypes, and only 3 of these animals had detectable serum viral RNA after challenge with wild-type dengue virus (DENV), suggesting protection of vaccinated animals to DENV infection. The vaccine induced statistically significant changes in 595 gene transcripts on days 1, 3, 5 and 7 as compared with baseline and placebo-treated animals. Genes involved in the type I interferon (IFN) response, including *IFI44*, *DDX58*, *MX1* and *OASL*, exhibited the highest fold-change in transcript abundance, and this response was strongest following double dose and subcutaneous (versus intradermal) vaccine administration. In addition, modules of genes involved in antigen presentation, dendritic cell activation, and T cell activation and signaling were enriched following vaccination. Increased abundance of gene transcripts related to T cell activation on day 5, and the type I IFN response on day 7, were significantly correlated with the development of high neutralizing antibody titers on day 30.

**Conclusions/Significance:**

These results suggest that early transcriptional responses may be predictive of development of adaptive immunity to TDV vaccination in cynomolgus macaques, and will inform studies of human responses to dengue vaccines.

## Author Summary

Dengue has become the leading cause of mosquito-borne virus infections worldwide. Despite considerable effort, development of a successful vaccine against dengue virus (DENV) has been challenging due to the co-circulation of the four DENV serotypes in endemic areas—to which humans develop distinct immune responses, and the increased risk of severe disease in those with pre-existing immunity to one serotype when they are infected with a different serotype. In this study, we investigated the responses in macaques to vaccination with the tetravalent, live-attenuated vaccine, TDV, by different doses and routes of vaccine administration. We identify changes in macaque gene expression that occurred in the days immediately following vaccination with TDV, a time-period that is difficult to study during natural infection. The gene expression response was characterized by features of the innate immune response to virus, notably the type I interferon response, and began the day after TDV vaccination. This response correlated with the development of neutralizing antibodies, which means that it might serve as an early indicator of a subsequent protective immune response to dengue vaccines.

## Introduction

Over the last 50 years, the incidence of dengue has increased 30-fold, and now more than half of the world’s population is at risk of dengue virus (DENV) infection [1]. Transmitted by *Aedes* mosquitos, DENV has become the leading cause of mosquito-borne viral infections worldwide, with an estimated 390 million infections occurring each year [1]. The outcome of infection ranges from an asymptomatic state to classic dengue fever (DF) and severe and potentially life-threatening dengue hemorrhagic fever (DHF) and dengue shock syndrome (DSS).

Each of the four antigenically distinct serotypes of dengue virus (DENV1 –DENV4) is capable of causing severe disease. While infection with one serotype provides long-lasting protection against re-infection with that serotype, cross-protective immunity is temporary and lasts only several months [2]. Furthermore, secondary infection with a heterologous serotype greatly increases the risk of developing severe disease [3,4]. While there is currently no licensed vaccine against dengue, there are several dengue vaccine candidates in development [5]. Takeda Vaccines’ Tetravalent Dengue Vaccine Candidate (TDV) (formerly DENVax, Inviragen) consists of a live attenuated DENV-2 strain (TDV-2) and three chimeric viruses containing the prM and E protein genes of DENV-1, -3 and -4 expressed in the context of the TDV-2 genome backbone (TDV-1, TDV-3, and TDV-4, respectively) [6,7]. TDV has been shown to be immunogenic and efficacious in animal models [8–10], generally well tolerated in humans [11], and is currently in phase 2 clinical trials.

Studies of dengue infection have revealed unique transcriptional signatures during the acute phase of infection that are associated with subsequent disease severity [12–16]. A recent study examined the role of the innate immune response in modulating the humoral immune response [16]. Understanding the mechanisms underlying the development of protection against dengue, and responses to dengue vaccination, may be useful in the further development of an effective vaccine against dengue. Host genome-wide transcriptional profiling provides a means to identify changes in gene expression occurring immediately following vaccination that may play a role in the development of protective immunity. This approach has revealed useful, clinically-relevant signatures following immunization with a variety of different vaccines [17–22].

In this study, we used transcriptional profiling to characterize early changes in gene expression in peripheral blood cells of cynomolgus macaques following vaccination with TDV, involving different doses (single dose or double dose on day 0), and routes (subcutaneous or intradermal) of vaccine administration. We compared changes in transcript abundance following TDV vaccination with those following infection with wild-type (wt) DENV in cynomolgus macaques. Gene transcript abundances were correlated with measurements of viral load and neutralizing antibody titer to identify markers predictive of vaccine immunogenicity.

## Methods

### Vaccines and viruses

TDV was generated from cDNA clone-derived DENV-2 VV45R virus, based on the DENV-2 PDK-53 genome [10], and from three chimeric strains based on DENV-2 PDK-53 expressing the prM and E genes of wt DENV-1, DENV-3 or DENV-4, as described previously [10,8]. For the neutralization antibody assays, we used the wt viruses from which the prM and E genes for each TDV virus were derived [9,11,23]. DENV-2 New Guinea C and DENV-4 Dominica/81 were used for viral challenge, generously provided by Dr. Stephen Whitehead (US National Institutes of Health, Bethesda, MD).

### Ethics statement

Animal work was conducted at the Charmany Instructional Facility of the School of Veterinary Medicine, University of Wisconsin-Madison, and at the Wisconsin National Primate Research Center. All animal procedures were approved by the University of Wisconsin-Madison’s Graduate School Animal Care and Use Committee, and the protocol, #G00634, in which these procedures are described, was approved on 10/22/2013. The regulations/guidelines to which animal care and the animal use protocol adhered are “The Guide for the Care and Use Of Laboratory Animals, 8th Edition”; the United States Department of Agriculture (USDA) Animal Welfare Act and Animal Welfare Regulations; US Public Health Service Policy on Humane Care and Use of Laboratory Animals; US Government Principles for the Utilization and Care of Vertebrate Animals Used in Testing, Research, and Training; and the USDA Policy Manual.

Monkeys were singly housed in standard stainless steel primate cages (Suburban Surgical, Chicago IL). All animals had visual and auditory contact with each other in the same room. They were fed twice daily with commercial chow (Harlan Teklad #2050, 20% protein Primate Diet, Madison, WI) and given a variety of fruit in the afternoons. In addition, we provided foraging activities and physical environmental enrichment at least weekly for both activities. Housing rooms were maintained at 65–75°F, 30–70% humidity and on a 12:12 light–dark cycle (ON: 0600, OFF: 1800). Standard veterinary analgesia was available to animals if necessary with either NSAIDs or opioid derivative drugs such as buprenorphine.

### Animals and study design

Thirty-five adult male, DENV-seronegative cynomolgus macaques from Vietnam were placed in quarantine for 30 days prior to the start of the study. Five groups of animals (n = 6 animals per group) received TDV either subcutaneously (SC) in 0.5 mL inocula or intradermally (ID) in 0.1 mL inocula using a needle-free injector (PharmaJet device) or needle and syringe (N&S), as outlined in Table 1. Detailed experimental methods appear in S1 Text.

**Table 1:** Groups of animals and vaccination regimen.

Group	Treatment	No. of animals	Immunizations	No. of doses^a^	Route/Device^b^
1	TDV	6	Day 0	Two	ID/PharmaJet Injector
				(Both arms)	
2	TDV	6	Day 0, Day 60	One	ID/PharmaJet Injector
				(One arm)	
3	TDV	6	Day 0, Day 60	One	ID/N&S
				(One arm)	
4	TDV	6	Day 0	Two	SC/PharmaJet Injector
				(Both arms)	
5	TDV	6	Day 0, Day 60	One	SC/PharmaJet Injector
				(One arm)	
6	PBS	5	Day 0, Day 60	One	ID/PharmaJet Injector
	wt DENV2/4	4	Day 90	(One arm)	SC/N&S

^**a**^ Number of doses: Animals in groups 1 and 4 received two doses of TDV on day 0, one in each arm. Animals in groups 2, 3 and 5 received one dose of TDV on day 0 and a second dose on day 60, in alternate arms.

^**b**^ ID, intradermal; SC, subcutaneous; N&S, needle & syringe.

### Serum viral RNA

Viral RNA in serum samples was measured using a quantitative reverse transcription-polymerase chain reaction (qRT-PCR) as described previously [9,23]. The limit of detection for the qRT-PCR of 3.6 log_10_ copies/mL was determined for each viral RNA standard by creating a standard curve consisting of nine replicates per dilution.

### Microneutralization assay

Heat-inactivated serum samples (56°C for 30 min) were tested for neutralizing activity using a viral immunofocus reduction microneutralization assay with an ELISpot reader (AID, San Diego, CA), as previously described [23]. Fifty percent of the average number of foci in the negative control serum defined the cut-off point (PRNT_50_). The serum dilution closest to the cut-off was recorded as the reciprocal neutralizing titer.

### Genome-wide transcript abundance analysis of peripheral blood

Total RNA was extracted using the PAXGene Blood RNA kits (Qiagen, Valencia, CA), and 500 ng of total RNA was amplified using the TargetAmp Aminoallyl aRNA amplification kit (Epicentre, Madison, CA). 8 ug of amplified RNA and 5 ug Universal Human Reference aRNA (Stratagene, La Jolla, CA) were labeled using Cy5 and Cy3 dyes, respectively, and hybridized to Human Exonic Evidence Based Oligonucleotide (HEEBO) microarrays. Details of the protocol have been described previously [15]. HEEBO microarrays, containing 44,544 probes, were printed by the Stanford Functional Genomics Core Facility. A detailed description of this probe set can be found at (http://microarray.org/sfgf/heebo.do). Microarray data were submitted to the Princeton University MicroArray database for subsequent analyses. Previous studies have shown that HEEBO arrays can accurately measure the abundance of gene transcripts in nonhuman primates [24–26]. Data were normalized by local background subtraction and a global mean normalization using regression correlation. Data were filtered to exclude probes that did not demonstrate a regression correlation of ≥0.6 between Cy5 and Cy3 signal over the pixels comprising the array element, and intensity/background ratio >2 in at least one channel. The microarray data are available at Gene Expression Omnibus (GEO accession number GSE72430).

### Statistical analysis

Changes in transcript abundance were calculated by subtracting the log_2_ abundance value at baseline (average of days -11 and -2) from each subsequent time-point (days 1, 3, 5, 7, etc.) for each animal. Baseline-transformed data were used for comparison between the different groups. Significance Analysis of Microarrays (SAM) was used to identify significantly differentially expressed genes between predetermined groups; genes and their transcripts were considered significant at a false-discovery rate (FDR) <5% and fold-change ≥1.3 [27]. The fold-change reported is the difference in the average relative abundance between each of the two groups in the comparison. Gene enrichment analysis was performed using the Database for Annotation, Visualization and Integrated Discovery (DAVID) [28]. DAVID keywords included Gene Ontology (GO) terms, biological pathways from the Kyoto Encyclopedia of Genes and Genomes (KEGG), and Swiss-Prot keywords. Keywords were considered significant when p<0.05 after Benjamini-Hochberg correction for multiple testing. Pathway analysis was performed using Gene Set Enrichment Analysis (Broad Institute) [29] and Blood Transcription Modules (BTMs) [17] on genes pre-ranked by SAM score or Spearman correlation coefficient. BTMs were considered significant at a FDR<5%. Differences in median transcript abundance levels of specific genes between immunization groups were tested using the Mann-Whitney U test. Spearman correlation was used to correlate relative transcript abundance of 15,705 variable genes (filtered for probes with an intensity/background >2 and present in at least 80% of the samples) with peak viral RNA abundance and duration of detectable viral RNA after vaccination, and DENV-2 specific neutralizing antibody titer and median neutralizing antibody titer (median PRNT_50_ of the 4 serotypes) on day 30.

## Results

### TDV-induced early changes in peripheral blood transcript abundance

**Fig 1:**
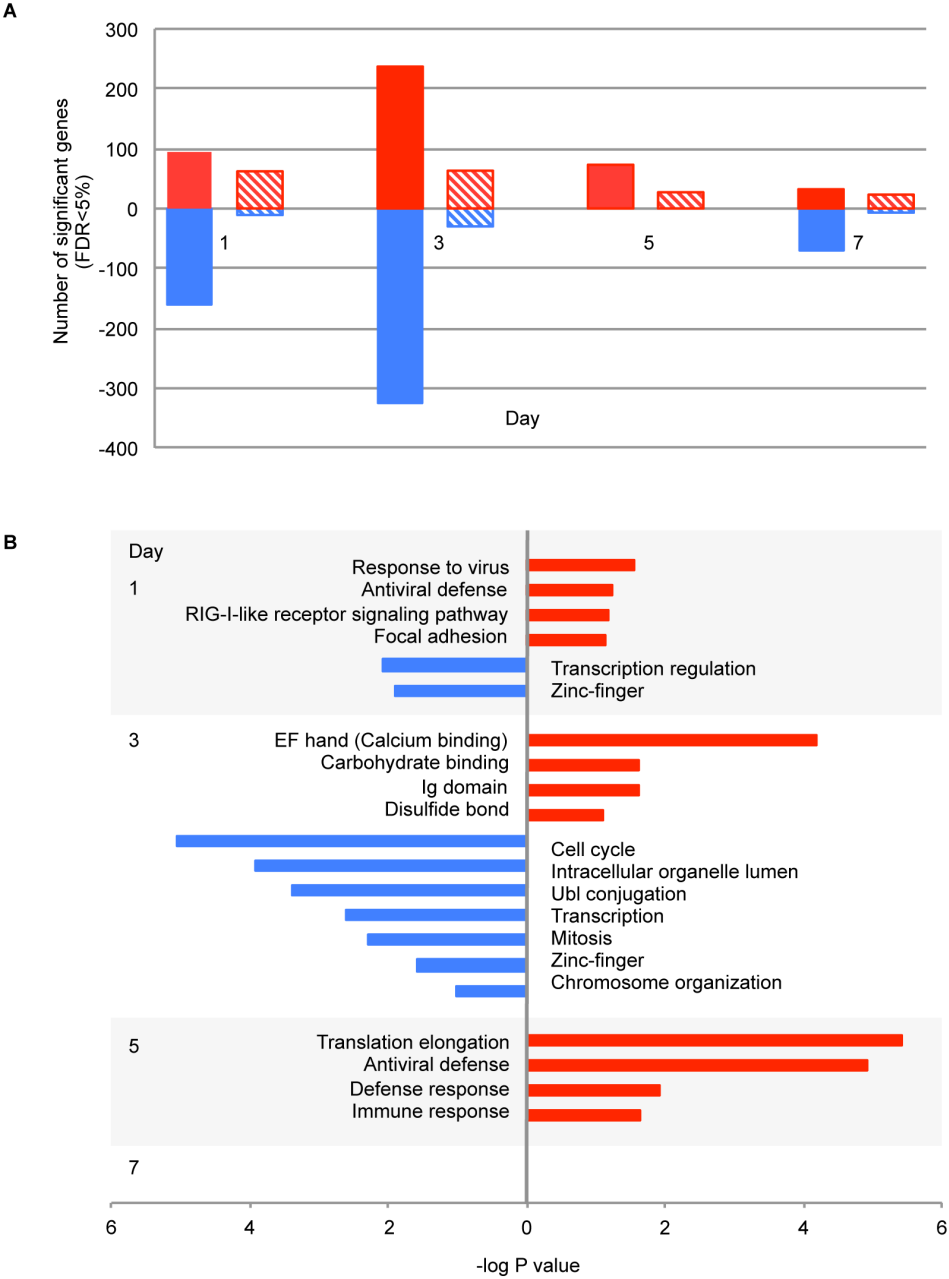
Changes in gene transcript abundance following TDV vaccination. Results from SAM analysis of transcripts from vaccinated animals compared with baseline, and vaccinated animals compared with placebo animals. (A) Numbers of differentially-expressed (DE) genes at each time-point. FDR<0.05; fold-change≥1.3 solid areas, fold-change≥1.5 dashed areas. Red bars indicate increased abundance; blue bars indicate decreased abundance. (B) Top GO terms associated with DE genes by day, p<0.05 after Benjamani correction for multiple testing. Strength of the association is shown on the abscissa. Red bars represent positively enriched GO terms; blue bars represent negatively enriched GO terms.

To characterize the overall response to TDV, we compared changes in transcript abundance by day in all 30 vaccinated animals against their pre-vaccinated baseline values. Five hundred and ninety-five genes were significantly differentially expressed (DE) in vaccinated animals compared with their own baseline on at least one day during the first week following vaccination with TDV (FDR<5%, fold-change≥1.3). Forty-six additional genes were differentially expressed in an unpaired SAM comparison between animals vaccinated with TDV and those that received the placebo (n = 5); these genes were included in this list (S1 and S2 Tables). A more stringent fold-change cut-off of 1.5 resulted in 217 DE genes (Fig 1A), and so we decided to use the 1.3-fold change cut-off in order to include a greater number of genes in the functional analysis. Functional annotation using DAVID identified GO terms associated with these gene sets that increased and decreased in abundance by day (S1 Table). Genes associated with the GO term “antiviral defense” (including *DDX58*, *EIF2AK2*, *ISG15*, *IRF7*, *MX1*, *IFI44*, *STAT1)* increased in abundance on day 1, but the association with this GO term was strongest on day 5 along with additional terms related to the response to vaccination (Fig 1B). Genes associated with cell cycle and transcription (including *CDC73*, *CCAR1*, *CNOT7*, *CREBZF*, *ZEB1*, and *FOS*) decreased in abundance on days 1 and 3. No GO terms were significantly enriched on day 7.

Transcripts for genes involved in viral recognition (*DDX58* (RIG-I) and *EIF2AK2* (PKR)), the type I interferon (IFN) response (*IRF7*, *OAS2* and *OASL*), the antiviral response (*MX1*), regulation of cytokine signaling (*STAT1*), and apoptosis (*XAF1)* exhibited the highest fold-changes in abundance compared with baseline (maximum fold-change 2.4) (S1 Table). The IFN stimulated gene (ISG) *ISG15* was up-regulated following TDV vaccination and is typically induced in response to type I IFN [30]. Secreted ISG15 acts on T and natural killer (NK) lymphocytes, in which it induces IFN-γ production, and has been shown to inhibit viral replication and suppress particle release of DENV-2 [30]. Transcripts for *TNFSF13B*, a gene associated with B cell activation [31] also increased in abundance following TDV vaccination.

**Fig 2:**
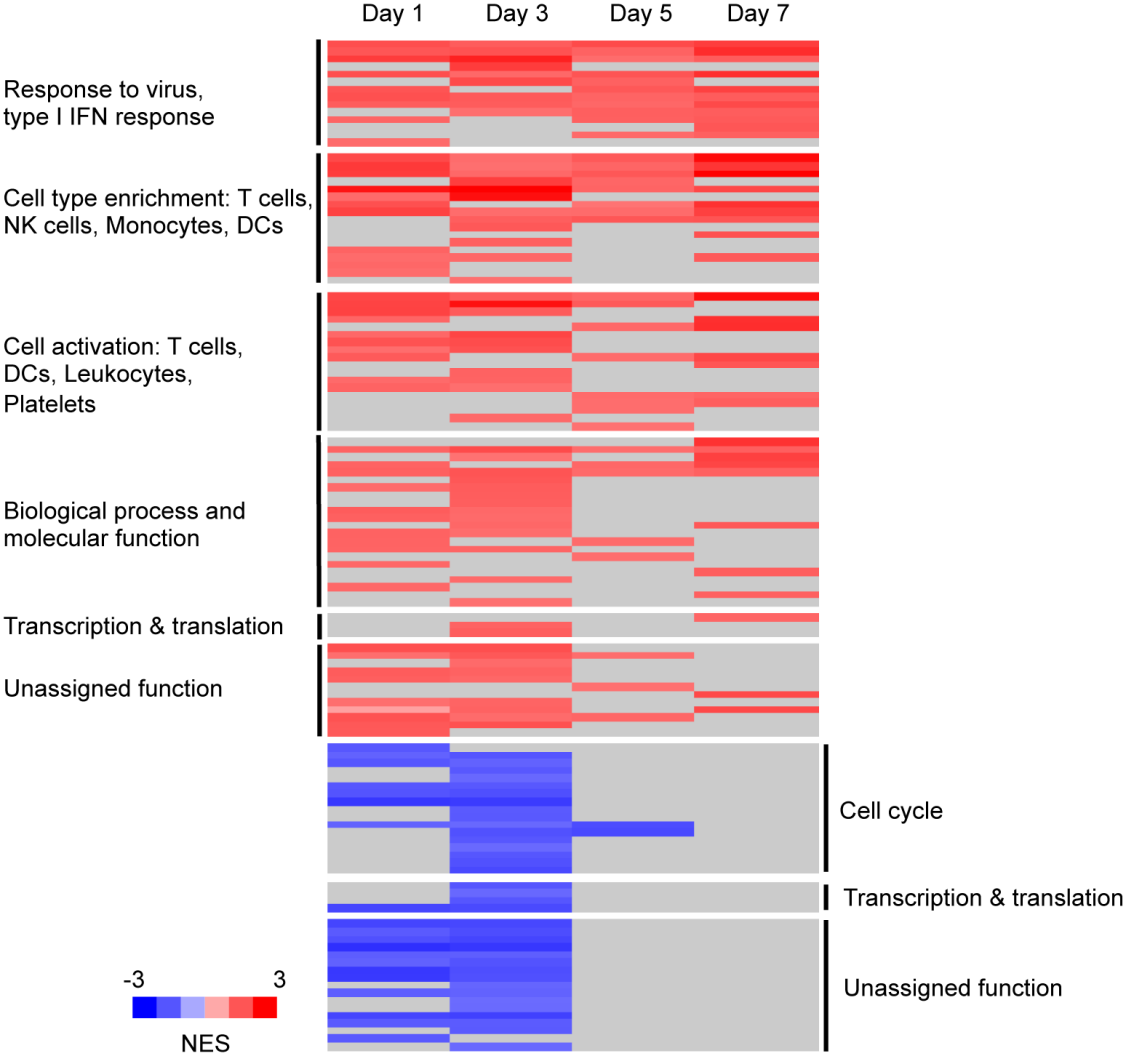
BTMs enriched over time following TDV administration across all animals. Modules are grouped (black vertical bars) by function and ordered by significance; only modules significantly enriched on at least one day are shown (FDR<5%). Positively enriched BTMs are shown in red, negatively enriched BTMs in blue, and no significant enrichment is shown in grey. NES, Normalized Enrichment Score [17].

To complement the gene-level analysis, we applied Gene Set Enrichment Analysis (GSEA) using the 334 Blood Transcription Modules (BTMs) constructed from publicly available microarray data specific to human blood [17], supplemented with 4 additional modules comprising cytokine-induced gene sets identified in our previous work [32] (S3 Table). Blood transcription modules also include sets of cell-type specific genes, which indicate the cell types that may contribute to the response based on the most differentially expressed genes [17]. Following vaccination, 86 BTMs were enriched for genes that increased in abundance (positively enriched), and 34 BTMs were enriched for genes that decreased in abundance (negatively enriched) (FDR<5%) (Fig 2, S4 Table). Positively enriched modules included those with functions associated with the innate immune response to viruses, antigen presentation, and activation of T cells, dendritic cells (DCs) and platelets. There was significant enrichment of genes specific to T cells, NK cells, neutrophils, monocytes and DCs (Fig 2, S4 Table). Negatively enriched modules were associated with mitosis, other aspects of the cell cycle and cell division, and inositol signaling. Inositol signaling has been shown to control the amplitude of type I IFN secretion and pDC activation, and usually inhibits cell activation [33].

### Effect of vaccination route and dose on the early transcriptional response

The transcriptional response in animals that received TDV by ID route (Groups 1 and 2) differed from those that received TDV via SC route (Groups 4 and 5) primarily by the temporal pattern of transcript abundance. Following ID immunization, most transcripts increased in abundance on day 1 (n = 142) and then decreased by day through day 7 (n = 0). On day 1 there was an increase in abundance of genes involved in lymphocyte activation (*THY1/CD90*, *CORO1A*, *LST1*, *STXBP2*, *TNFSF13* and *TNSF13B*), and viral recognition and the antiviral response (*DDX58*, *EIF2AK2*, *ISG15*, *IRF7*, *IRF9*, *IFI44*, *STAT1*, *OASL*, *OAS2*, *IFITM3* and *MX1*), which persisted on days 3 and 5. Following SC immunization, transcript abundance increased over time from day 1 (n = 18), 3 (n = 157), to 5 (n = 1460), and then decreased by day 7 (n = 17). On day 5 following SC immunization, there was an increase in abundance of genes related to T and B cell activation and differentiation (including *BCL2*, *CD28*, *CD47*, *IL15*, *TNFSF13B* and *KLRK1*), regulation of cytokine production, and the JAK/STAT signaling cascade (*JAK2*, *IL6ST*, *STAT1*, *STAT2*, *STAT4*, and *SOC2S*). On day 7, only genes involved in viral recognition and the antiviral response were enriched (*DDX58*, *EIF2AK2*, *ISG15*, *IFI44*, *STAT1*, *OAS2*, *OASL*, *XAF1* and *MX1*). No significant differences in gene enrichment were observed by direct comparisons of relative transcript abundance levels between animals that received ID vaccination versus SC vaccination.

**Fig 3:**
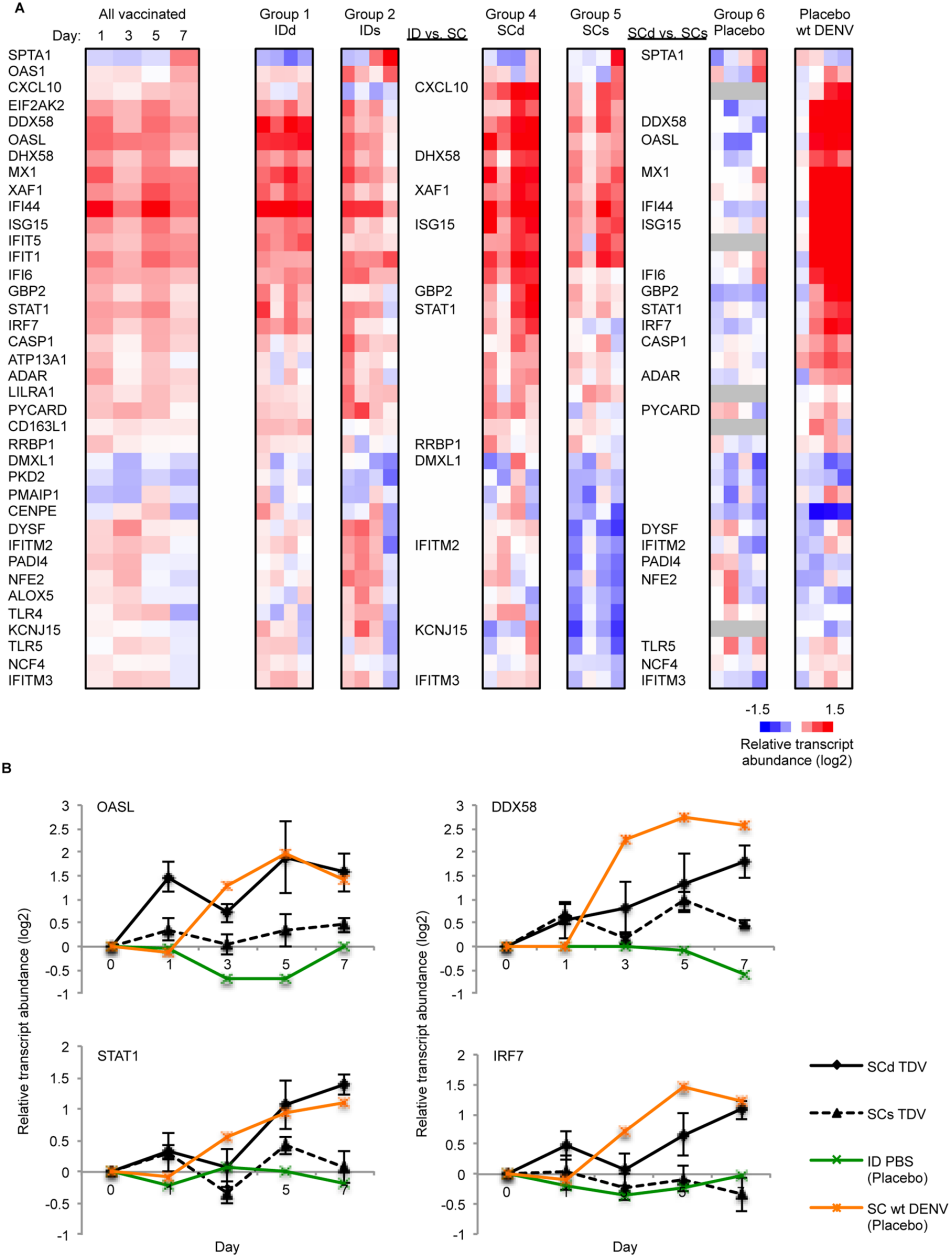
Antiviral/type I IFN response following TDV vaccination. A) Median abundances of 38 transcripts over time (days post-vaccination) in all vaccinated animals, and by group. Red indicates an increase in transcript abundance; blue indicates decreased transcript abundance (FDR<0.05, fold-change≥1.3). Genes listed when significant in comparison between intradermal (ID) and subcutaneous (SC) groups, and SC double dose (SCd) and SC single dose (SCs) groups (p<0.05, ranksum). Placebo group, and placebo animals challenged with wt DENV, shown for comparison. B) Median relative transcript abundance over time by vaccination group. Bars indicate standard error.

In all groups, the highest fold-change in gene abundance following vaccination was observed for genes involved in the antiviral and type I IFN response. To investigate how the observed responses were modulated by dose and route of vaccine administration we created a master list of 379 genes involved in the antiviral/type I IFN response by combining the genes present in relevant BTMs. Overall, 38 of these genes were significantly differentially expressed following TDV vaccination (Fig 3A). A greater number of these genes were significantly differentially expressed in the groups of animals that received SC immunization (single or double dose) compared with those that received ID immunization (S1 Fig), suggesting that SC immunization results in a broader antiviral/type I IFN response than ID immunization. To evaluate whether dose or route of vaccine administration altered the strength of the antiviral/type I IFN response, we compared expression of each gene by vaccination group over time (Mann-Whitney U test, p<0.05). Abundances for 11 gene transcripts differed significantly between SC and ID vaccination (single and double dose groups combined); the majority had higher levels following SC vaccination (Fig 3A). For dose, we focused on the SC double (SCd) and single (SCs) dose groups (groups 4 and 5) since a greater number of changes in transcript abundance were observed in these groups compared with the ID groups (groups 1–3), and because SC administration is a route used for human trials of TDV. Following double dose SC vaccination, 20 genes had significantly higher transcript abundances during at least one time-point compared with single dose SC vaccination (Fig 3A and 3B). Most of these differences occurred on day 7 (15 genes), suggesting that increasing the dose of TDV elicits a stronger and more prolonged response for these genes.

### Transcriptional response to wild-type DENV is most similar to the response to double dose SC TDV

**Fig 4:**
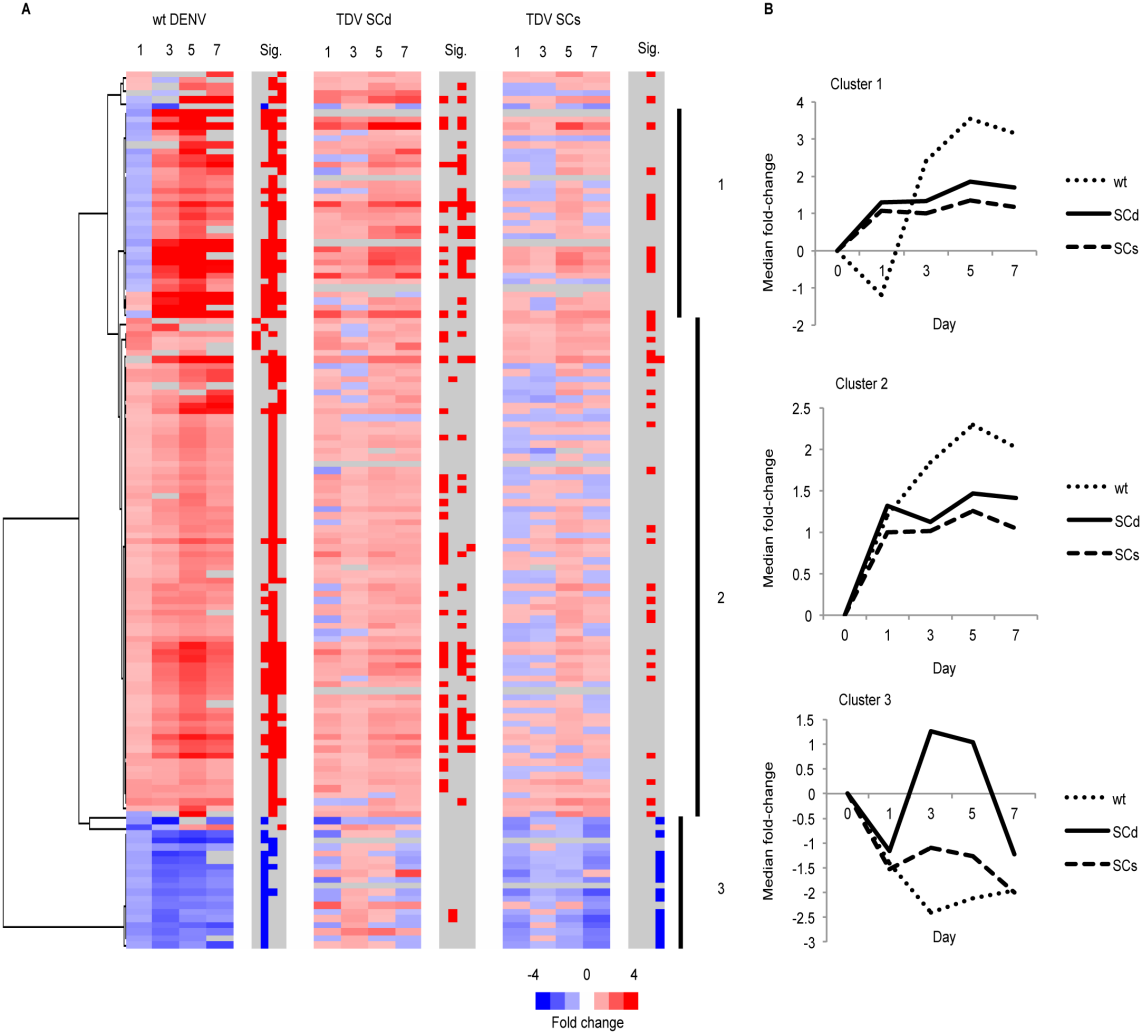
Comparison of changes in transcript abundance following wt DENV infection and TDV immunization. A) Hierarchical clustering of 135 genes whose transcripts differ in abundance over time following wt infection of sham-immunized animals (FDR<0.05, fold-change≥1.3). Median fold-change in transcript abundance is shown over time for each group; statistical significance (FDR<5% and fold-change ≥1.3) is indicated in right-hand grey column. Red indicates increased abundance; blue indicates decreased abundance. Black vertical bars delineate gene clusters 1, 2 and 3. B) Median fold-change of transcript abundance for each gene cluster by group. wt DENV, wild-type dengue virus; SCd, subcutaneous double dose; SCs, subcutaneous single dose.

Wild-type (wt) challenge of placebo animals with DENV-2 (3 animals) or DENV-4 (1 animal) by SC inoculation led to significant changes in the abundance of 135 genes over time (Fig 4A). The smaller number of significant genes may reflect the smaller sample size of 4 animals. Unsupervised hierarchical clustering of these genes revealed 3 clusters (Fig 4B, S5 Table). Clusters 1 and 2 were both significantly enriched for genes we previously identified as induced by type I IFN (p<1E-10 for each cluster) [32], though only genes from cluster 1 were significantly associated with GO terms, including “response to virus” (p = 1.2E-8), “immune response” (p = 0.0001), and “GTP-binding” (p = 0.04). Cluster 3 was not associated with any GO terms. While abundance of transcripts in clusters 1 and 2 increased following both infection with wt DENV and vaccination, the magnitude of response was much greater following wt virus infection, as seen in median fold-change over time of transcripts for genes in each cluster (Fig 4B). Fold-change peaked at 19.9 (*ISG15*) following wt DENV infection compared with 5.7 (*IFI44*) following double dose SC TDV vaccination. Wt virus caused a decrease in transcript abundance for genes in Cluster 3. Proteins expressed by genes in this cluster include IL1-R2, a negative regulator of IL-1 signaling [34], and CRISPLD2, a serum protein produced by monocytes, NK cells, and T cells in response to stimulation by LPS and other PAMPs, including poly (I:C) [35,36].

### TDV-2 viral load correlates with DENV-2 neutralizing antibody titer and features of the early response

Over half of the animals (17/30) had detectable viral RNA (vRNA) (range: 3.8–5.6 log_10_ copies/mL) between days 5 and 14 following primary immunization (mean duration, 5 days) (Table 2). Only TDV-2 virus RNA was detected in all cases, consistent with previous studies of TDV immunization [9,11]. Most animals that received TDV by SC (single and double dose) and ID (double dose only) routes had vRNA, compared with only 1 animal that received TDV by single dose ID route (Table 2). After challenge with wt DENV, only 3 of the vaccinated animals developed detectable vRNA, compared with all placebo (sham-immunized) animals (range: 3.7–5.8 log_10_ copies/mL; mean duration, 4 days), suggesting that TDV protected against wt DENV challenge (Table 2).

**Table 2:** Viral load (range) after TDV vaccination and DENV-2/DENV-4 challenge.

Group	Treatment	Dose	Viral load after TDV immunization (log10 copies/ml)					Viral load after DENV-2/DENV-4 challenge (log10 copies/ml)					
			d5	d7	d10	d12	d14	d91	d93	d95	d97	d99	d101
1	TDV	Two	-	3.9–4.8 (3/6)	4.5–5.3 (4/6)	3.9–5.2 (5/6)	3.8–5.1 (3/6)	4.0 (1/6)	-	4.5 (1/6)	-	-	-
	ID/PhJ	(0, 0)											
2	TDV	One	-	-	-	-	-	-	-	-	3.8 (1/6)	-	-
	ID/PhJ	(0, 60)											
3	TDV	One	-	-	3.8 (1/6)	3.8 (1/6)	3.8 (1/6)	-	-	-	-	-	-
	ID/N&S	(0, 60)											
4	TDV	Two	3.8 (1/6)	3.8–5.0 (5/6)	3.7–5.3 (5/6)	4.0 (1/6)	-	-	-	-	-	-	-
	SC/PhJ	(0, 0)											
5	TDV	One	-	3.9–5.6 (3/6)	3.8–5.4 (4/6)	4.3–5.0 (3/6)	3.7–4.2 (2/6)	-	-	-	-	-	-
	SC/PhJ	(0, 60)											
6	PBS	One	-	-	-	-	-	4.3 (1/6)	4.1–4.8 (5/5)	4.3–5.8 (4/5)	4.8–5.2 (3/5)	3.7 (1/5)	4.7 (1/5)
	SC/PhJ	(0, 60)											

Number of animals from each group with detectable viral RNA shown in parentheses. No vRNA was detected in any animals on days 0, 3, 53, 64, 67, 88, 102, and 104.

Correlation of transcriptional responses (relative transcript abundance by day) to the duration and peak of viral load revealed significant correlations with genes involved in B and T cell activation and differentiation on day 5 post-immunization. Genes included *IRF4* (Spearman’s *ρ* = 0.70, p = 1.76E-05) and *ITK* (Spearman’s *ρ* = 0.58, p = 0.0009), which encode intracellular tyrosine kinases involved in the regulation of T cell development and differentiation, and *CCR6* (Spearman’s *ρ* = 0.52, p = 0.006), which encodes a beta chemokine receptor important for B-lineage maturation and antigen-driven B-cell differentiation. Both the duration and peak of TDV-2 virus load were positively correlated with the development of higher DENV-2 neutralizing antibody titers on day 30, when they were highest (*r*^*2*^ = 0.42 and 0.40, respectively) (S2 Fig).

### Features of the innate immune response to TDV correlate with neutralizing antibody titer

**Fig 5:**
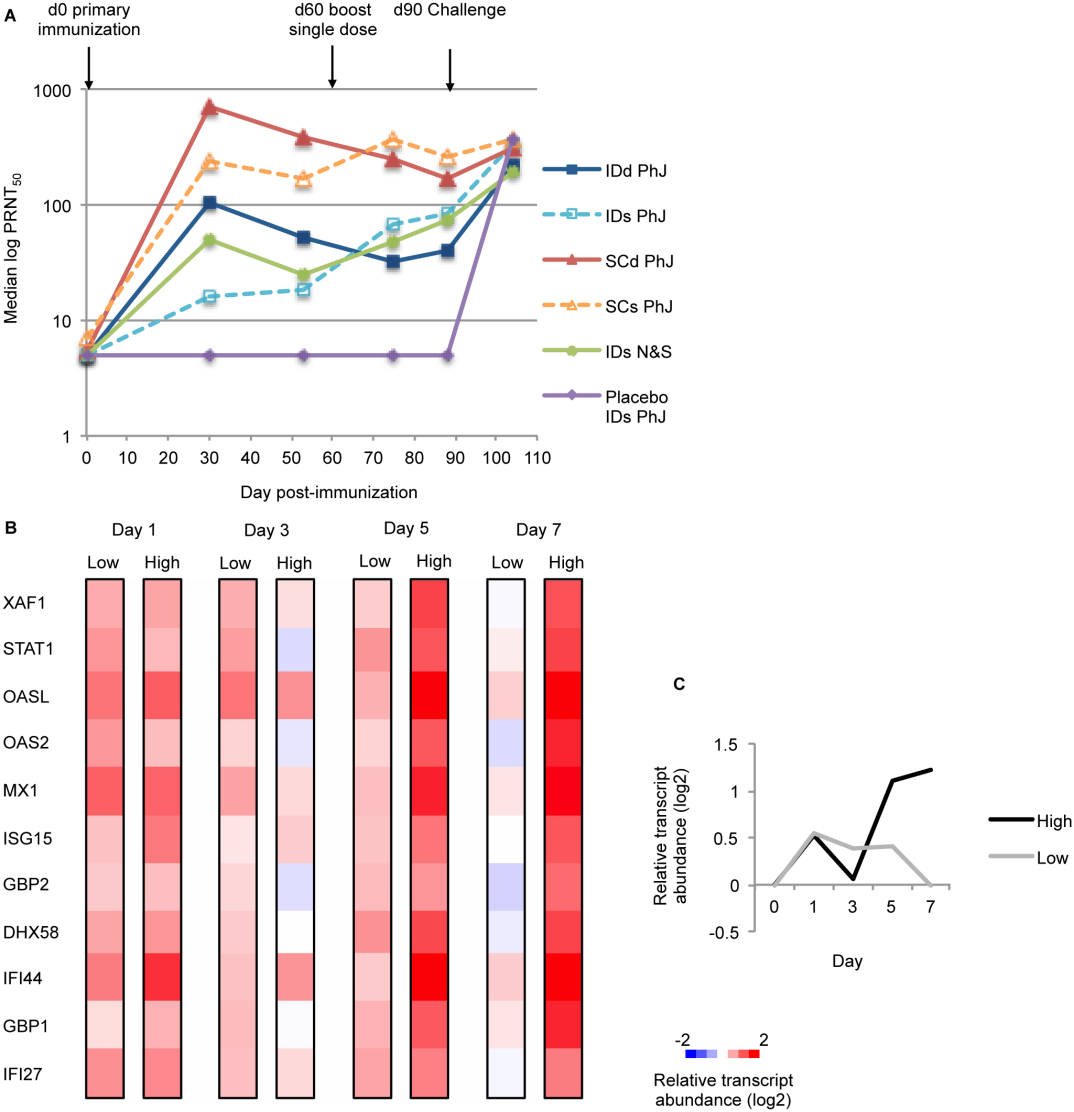
Correlation of viral load and relative transcript abundance to neutralizing antibody titer, by animal. A) Median neutralizing antibody titer (median log PRNT_50_) over time, by vaccination group. Colors represent different vaccination groups. B) Relative transcript abundance for the 11 type I IFN genes significantly positively correlated with neutralizing antibody titer on day 30. Median expression in 5 lowest responders compared with median expression in 5 highest responders over time. C) Median relative transcript abundance of the 11 type I IFN genes over time in high responders (black line) and low responders (grey line). IDd, intradermal double dose; IDs, intradermal single dose; IDs N&S, intradermal single dose needle and syringe; SCd, subcutaneous double dose; SCs, subcutaneous single dose.

All animals vaccinated with TDV developed a neutralizing antibody response to each of the four dengue serotypes (S3 Fig). Median neutralizing antibody titer (median PRNT_50_) was calculated to represent an overall response to all serotypes of the tetravalent vaccine. Median PRNT_50_ differed between the groups, and was highest for animals administered SC TDV (Fig 5A). Median titers were higher in animals that received double dose TDV rather than a single dose before boost (days 30 and 53), but were similar post-boost (days 75 and 88), and did not change significantly after challenge with wt DENV (Fig 5A and S3 Fig). Six hundred and thirteen genes were significantly correlated with median PRNT_50_ on day 30 during at least 1 time-point following vaccination (Spearman’s rank correlation coefficient, p<0.01). The strongest correlations were seen with genes related to the type I IFN response on day 7, including *DHX58*, *OASL*, *GBP1*, *GBP2*, *IFI27*, *XAF1* and *STAT1* (Spearman’s *ρ*: 0.58–0.64, p<0.0007). On day 5, transcript abundance for *KLRC3* (killer cell lectin-like receptor subfamily *c*, member 3), which is expressed primarily in NK cells and involved in T cell responses, was positively correlated with median PRNT_50_ on day 30 (Spearman’s *ρ* = 0.52, p = 0.004).

Forty-two of these genes also showed significant changes in abundance following vaccination (S6 Table), and 11 were involved in the antiviral/type I IFN response described above. The temporal transcript abundance pattern of these 11 genes differed between all ‘low responders’ (n = 5 animals whose PRNT_50_ on day 30 was less than 4-fold greater than baseline) and ‘high responders’ (n = 5 animals with the highest PRNT_50_ on day 30) (Fig 5B and 5C). The type I IFN response is induced in response to viral sensing, but the transcript median relative abundance of this type I IFN gene set was not significantly correlated with either the extent or duration of viremia (peak viral load *r*^*2*^ = 0.34, duration of viremia *r*^*2*^ = 0.32) (S4 Fig). Pathway analysis using BTMs of genes ranked by correlation score also showed that modules of genes related to T cell activation and differentiation were enriched on day 5, and the type I IFN response was enriched on day 7, supporting these findings (S7 Table).

In most cases, the dominant neutralizing antibody response was directed against serotype 2 (S3 Fig), as seen previously [9,11]. Comparison of transcriptional responses by day to DENV-2 specific neutralizing antibody titers pre-boost on day 30 revealed a positive correlation with the relative transcript abundance for genes involved in T cell proliferation and activation on day 5. These genes included *PRKCQ*, which encodes the enzyme, protein kinase C theta, required for T cell activation (Spearman’s *ρ* = 0.6, p = 0.001), and *IL6ST*, which encodes a cytokine signal transducer (Spearman’s *ρ* = 0.62, p = 0.0004). When serotype responses were examined separately, 37 transcripts were significantly correlated with the day 30 neutralizing antibody response for at least 3 of the 4 DENV serotypes. While there were no significant gene ontologies, positive regulation of apoptosis was a common signature, and included the genes *XAF1*, *BARD1*, *GCH1*, *MAGED1*, *PRKCA*. Four of these transcripts, which are all involved in the antiviral/type I IFN response, were also significantly enriched following TDV vaccination (*DHX58*, *IFI27*, *ISG15*, and *XAF1*).

## Discussion

Understanding the mechanisms that underlie the development of protective immunity against dengue infection may assist in the development of an effective vaccine against dengue. In this study, we used genome-wide transcriptional profiling to identify early transcriptional responses to vaccination that may act as predictors of an effective vaccine response. All animals vaccinated with TDV developed neutralizing antibodies to each of the 4 dengue serotypes, and in most cases the dominant antibody response was to serotype 2, as seen previously [9,11]. TDV-2 was the only strain with vRNA detected after vaccination, and the abundance of TDV-2 vRNA was positively correlated with the development of DENV-2 neutralizing antibodies. Furthermore, we observed changes in transcript abundance for genes related to T cell activation on day 5 that were positively correlated with the presence of TDV-2 vRNA on days 5–14, and the development of higher DENV-2 specific neutralizing antibody titers on day 30, suggesting that TDV-2 is more immunogenic than TDV-1, TDV-3 and TDV-4, as seen in humans [11].

Changes in abundance of transcripts related to the antiviral and type I IFN response were the most notable features of the response to TDV vaccination These changes were strongest following subcutaneous compared with intradermal vaccination, and double dose administration on day 0 compared with single dose. The type I IFN response is well described for natural DENV infection [12–16], and appears to be associated with vaccination using live attenuated viruses. The live, attenuated yellow fever vaccine (YF-17D) [17,19,22] and influenza vaccine (LAIV), both resulted in a type I IFN response, but trivalent inactivated influenza vaccine (TIV) did not, suggesting that viral replication may also increase immunogenicity of LAIV vaccines [20]. It has recently been shown that IFN-α production by pDCs following dengue infection requires active viral replication in neighboring infected cells, but is triggered by internalization in pDCs of non-infectious viral components [37]. In this study, animals that experienced higher viral loads tended to have higher abundances of gene transcripts related to the type I IFN response, but the correlation was not significant. While we cannot conclude from these data that a stronger IFN response clears the virus, studies of dengue infection in humans and mice indicate that the innate response is important for controlling viral replication and pathology, and that stronger IFN responses are beneficial to the host.

Type I IFNs (IFN-α/β) are pleiotropic cytokines that play important roles in both innate and adaptive immune responses [32,38–40]. During dengue infection, viral recognition of dsRNA by pattern recognition receptors and the cytoplasmic helicases retinoic-acid-inducible gene I (RIG-I) and melanoma differentiation-associated gene 5 (MDA5), leads to an intracellular signaling cascade responsible for the production of IFN-α/β[38]. IFN-α/β activates the JAK/STAT pathway, inducing expression of many interferon stimulated genes (ISGs). Transcripts for several genes in this pathway became more abundant following TDV vaccination, including *DDX58* (RIG-I), *EIF2AK2* (PKR), *STAT1*, *IRF7*, *OASL* and *OAS2*, *MX1*, *IFI44*, *ISG15*, and *XAF1*. The magnitude of the type I IFN response was much greater following infection with wt DENV, which might reflect greater replication and increased immunogenicity of the wt virus compared with vaccine viruses. However, the response observed in NHPs following wt DENV infection was more subtle compared with natural infection in humans [15], consistent with the lack of pathology in non-human primates following dengue infection.

The type I IFN response that dominated the response to TDV was modulated by both dose and route. After ID immunization, most of the increases in abundance of genes related to the type I IFN response and lymphocyte activation occurred on day 1, whereas after SC immunization these genes increased in abundance on days 5 and 7. ID immunization targets Langerhans and dermal dendritic cells, and macrophages in the epidermis and dermis. The dermis is highly vascularized, and skin DCs, antigen presenting cells, and monocytes and neutrophils recruited from the peripheral blood, transport antigens and vaccine components to draining lymph nodes, limiting transfer to the peripheral blood circulation [41]. The earlier type I IFN transcriptional response, and reduced detectable viremia, following ID immunization may be due to the skin’s resident immune cells and faster clearance of the vaccine. The stronger, and later response in a subset of type I IFN genes following subcutaneous immunization, particularly double dose administration, may have resulted from the larger volume and/or dose of vaccine inoculum (S1 Materials and Methods), and prolonged persistence of the vaccine virus in the adipose tissue of the SC layer [41,42]. Numerous toll-like receptors (TLRs) are expressed by the skin’s immune cells, and by fibroblasts, adipocytes and macrophages of the subcutaneous tissue. TLRs recognize microbial pathogens and trigger signaling cascades involved in both innate and adaptive immune responses. Transcripts encoding TLR4 and TLR5 increased in abundance following TDV vaccination, yet there were no significant differences in expression between ID and SC routes.

Increased abundances of transcripts for genes involved in the type I IFN response were correlated with the development of higher neutralizing antibody titers on day 30, suggesting that type I IFN may play a role in the activation of B cells. The sequence of events linking type I interferon responses to neutralizing antibody titer in our study is not known, but the importance of type I IFN for the activation of the production of antibody by B cells in other systems has been described previously [43–45]. Deal *et al*. demonstrated that pDC-derived type I IFN was required to activate B cells for production of virus-specific antibodies in human *in vitro* and mouse *in vivo* models of rotavirus infection [46]. While neutralizing antibody titers are the primary measure of immunity to dengue virus infection and the response to vaccination, they do not always reflect protection. In fact, T-cell responses may also play an important role in immunity to dengue [47]. The live-attenuated tetravalent chimeric yellow fever-dengue vaccine (CYD23) resulted in no protection against DENV-2 in a phase 2b efficacy trial, and lower efficacy (35%) in a phase 3 trial, despite high neutralizing antibody titers against all four serotypes [48,49]. This may be due to the absence in CYD23 of the dengue backbone that harbors important epitopes targeted by CD8T^+^ T-cells [47]. In our study, increased abundance of genes related to T cell activation on day 5 correlated with the concomitant presence of TDV-2 vRNA (days 5–14) and subsequent DENV-2 specific neutralizing antibody titers on day 30, suggesting a TDV-2 specific adaptive response. We were unable to correlate the transcriptional response with the development of cell-mediated immune responses, as these were only measured for animals in Group 4 (SCd PhJ) [23]. In these animals, TDV induced CD4+ and CD8+ T cells that targeted DENV-2 NS1, NS3 and NS5 proteins and that cross-reacted with DENV-4 NS3 and NS5 proteins [23]. Additional studies will be useful for evaluating links between the early transcriptional response and the development of cell-mediated immune responses.

This study focused on the response to TDV in non-human primates, which serve as important animal models for understanding vaccine responses and efficacy [50]. The responses we observed are similar to those elicited by DENV infection in humans; however, these non-human primates do not develop signs of disease following DENV infection. Characterization of gene expression changes that occur in humans vaccinated with dengue vaccines will be valuable for investigating the role of specific cell types in shaping innate and adaptive immunity.

## Supporting Information

S1 Text Supporting information on viruses, animals and study design. (DOCX)

S1 Table Genes differentially expressed after TDV vaccination in 30 animals compared to baseline. (XLSX)

S2 Table Baseline-transformed (average of d-11 and d-2) relative transcript abundances (log2) by animal for 595 differentially expressed genes after TDV vaccination. (XLSX)

S3 Table Additional modules of IFN-induced gene sets. (XLSX)

S4 Table Blood transcript modules enriched following vaccination (FDR<5%). (XLSX)

S5 Table Fold-change over time of significant genes in Clusters 1–3 for animals in the placebo (PBS) group following wt DENV challenge. (XLSX)

S6 Table Overlap of genes with significant changes in transcript abundance following vaccination and correlated with median neutralizing antibody titers on day 30. (XLSX)

S7 Table Enriched blood transcript modules from genes ranked by Spearman correlation coefficient *r* (FDR<5%). (XLSX)

S1 Fig The antiviral/type I IFN response following TDV vaccination. Unsupervised clustering of median expression of 282 genes (379 genes, filtered for reliably measured transcript abundance in 2 out of 3 samples) over time in all vaccinated animals, and median expression by group. Red indicates an increase in transcript abundance, blue indicates a decrease in transcript abundance (FDR<0.05, fold-change≥1.3). Significance by group marked by grey column at the right of each heatmap, with red (increase in abundance) and blue (decrease in abundance). Placebo vaccination and placebo recipient challenge with wt DENV shown for comparison.
(PDF)

S2 Fig Correlation of DENV-2 neutralizing antibody titer (PRNT_50_) on day 30 with (A) duration of viremia; and (B) peak viral load. (PDF)

S3 Fig Geometric mean neutralizing antibody titer (PRNT_50_) over time for each treatment group. (PDF)

S4 Fig Correlation between abundance of type I IFN genes and (A) duration of TDV-2 viremia and (B) peak TDV-2 viral load. (PDF)
