# Supplementary material for: Early Transcriptional Signatures of the Immune Response to a Live Attenuated Tetravalent Dengue Vaccine Candidate in Non-human Primates
Source: PLoS Negl Trop Dis. 2016 May 23;10(5):e0004731. doi: 10.1371/journal.pntd.0004731 (PMC4877054; doi:10.1371/journal.pntd.0004731)
Supplement: S1 Text — (DOCX) [file pntd.0004731.s001.docx]

**S1 (Supporting) Text**

**2.1 Vaccines and viruses**

The wt viruses were grown in Vero cells (challenge viruses) or C6/36 cells (virus strains used in the neutralization assay) in Dulbecco`s modified minimal essential medium (DMEM) containing, fetal bovine serum (FBS) penicillin-streptomycin.

**2.2 Animals and study design**

The vaccine formulation was adjusted to be the same dose (2x10^4^ pfu TDV-1, 5x10^4^ pfu TDV-2, 1x10^5^ pfu TDV-3, 3x10^5^ pfu TDV-4) for both SC and ID inocula. A control group (n=5 animals) received 0.1 mL PBS via ID route using the PharmaJet device. Animals received two doses (same route) of TDV either on the same day (Day 0) or sixty days apart (Table 1). On day 90 after primary vaccination, 3 animals from each group were challenged with 10^5^ PFU wt DENV-2 (New Guinea C strain) or 10^5^ PFU wt DENV-4 (Dominica/81 strain) by SC route using N&S. Serum samples were collected on days 0, 3, 5, 7, 10, 12, 14, 53, 64, 67, 88 post-immunization to quantify TDV viral RNA (vRNA) and days 91, 93, 95, 97, 99, 101, 102 and 104 to analyze wt DENV vRNA after challenge. Serum samples were also collected on days 0, 30, 53, 75, 88 and 104 to determine the levels of neutralizing antibody directed against each serotype. Blood samples for microarray analysis were collected on days -11 and -2 before vaccination; days 1, 3, 5 and 7 after vaccination, and days 91, 93, 95 and 97 after wt DENV challenge. Samples were stored at -80C prior to processing.
