# Supplementary figures and images for: Early Transcriptional Signatures of the Immune Response to a Live Attenuated Tetravalent Dengue Vaccine Candidate in Non-human Primates
Source: PLoS Negl Trop Dis. 2016 May 23;10(5):e0004731. doi: 10.1371/journal.pntd.0004731 (PMC4877054; doi:10.1371/journal.pntd.0004731)

S1

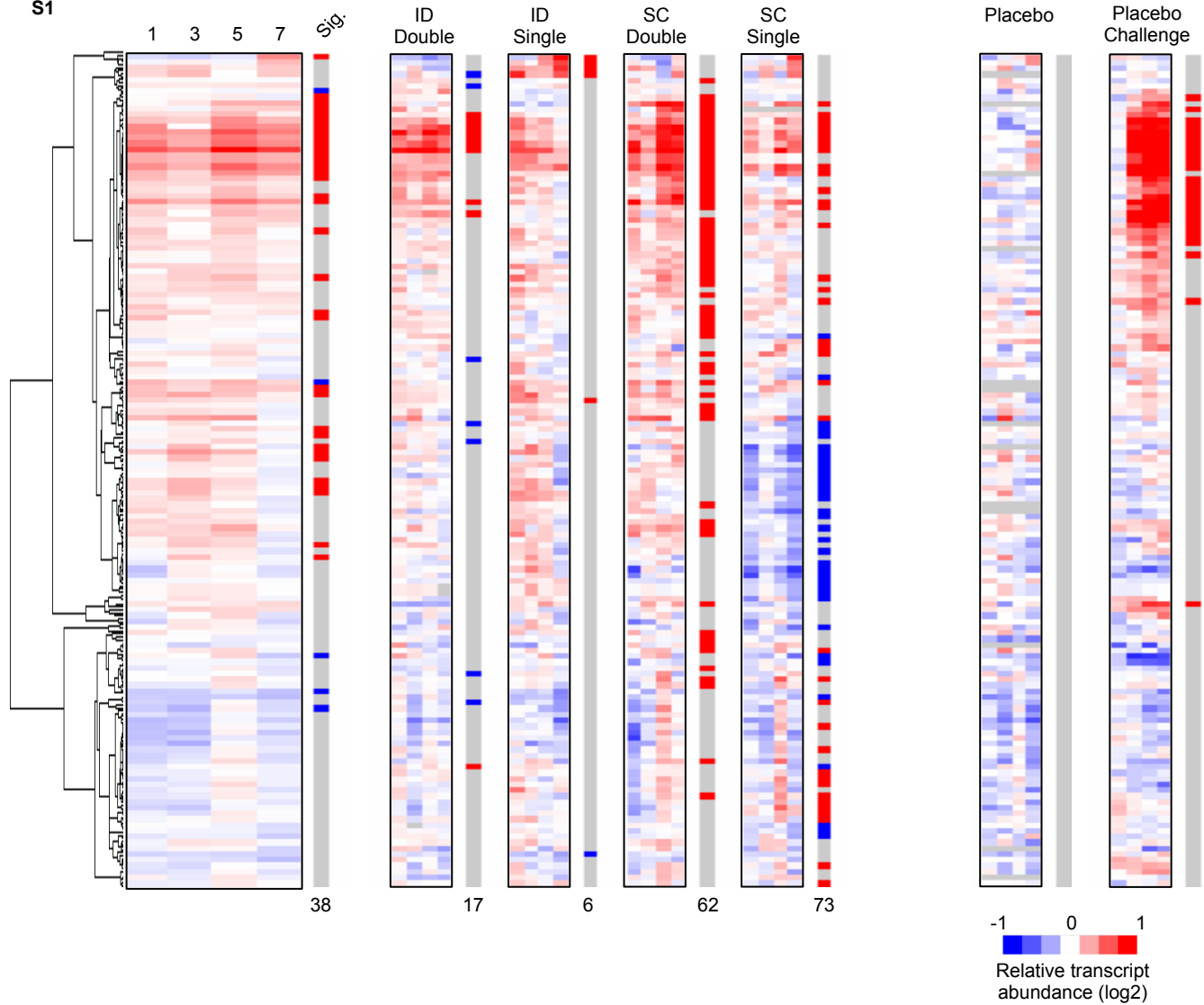

Supplement: S1 Fig — Unsupervised clustering of median expression of 282 genes (379 genes, filtered for reliably measured transcript abundance in 2 out of 3 samples) over time in all vaccinated animals, and median expression by group. Red indicates an increase in transcript abundance, blue indicates a decrease in transcript abundance (FDR<0.05, fold-change≥1.3). Significance by group marked by grey column at the right of each heatmap, with red (increase in abundance) and blue (decrease in abundance). Placebo vaccination and placebo recipient challenge with wt DENV shown for comparison. (PDF) [file pntd.0004731.s009.pdf]

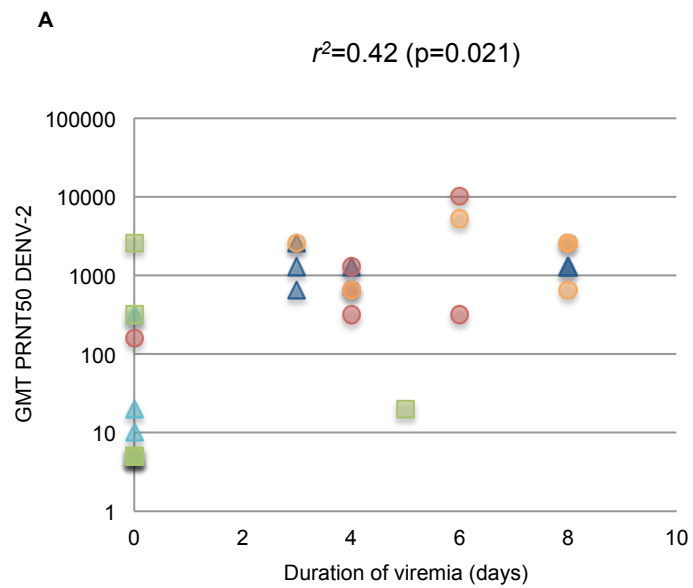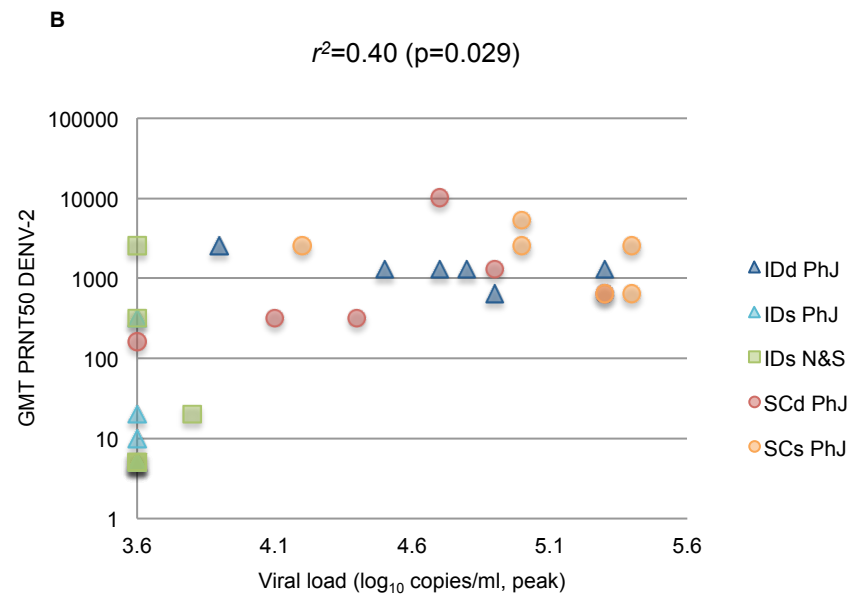

Supplement: S2 Fig — (PDF) [file pntd.0004731.s010.pdf]

ID double dose PhJ

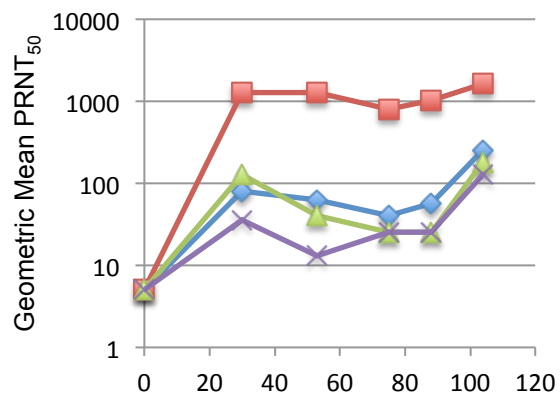

ID single dose PhJ

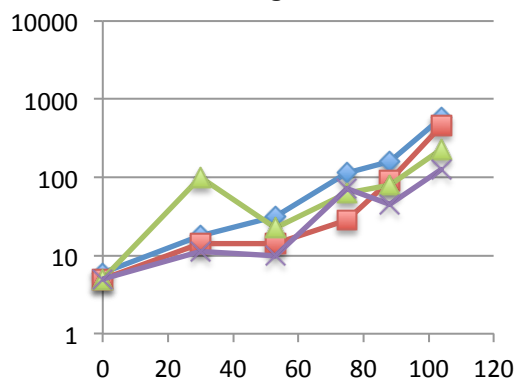

SC double dose PhJ

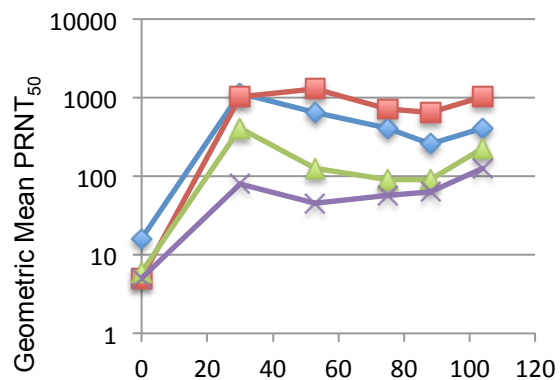

SC single dose PhJ

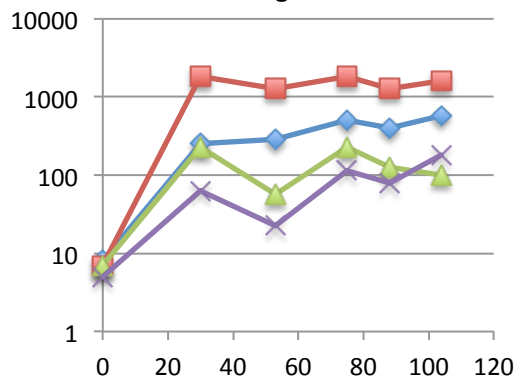

ID single dose NS

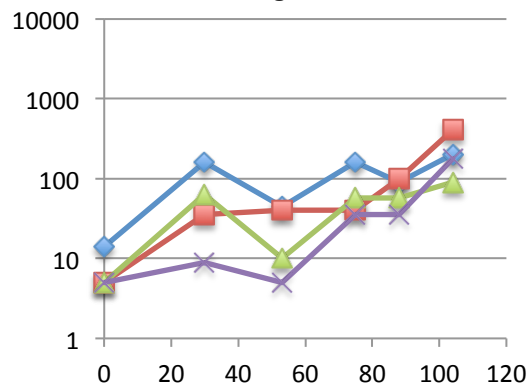

Supplement: S3 Fig — (PDF) [file pntd.0004731.s011.pdf]

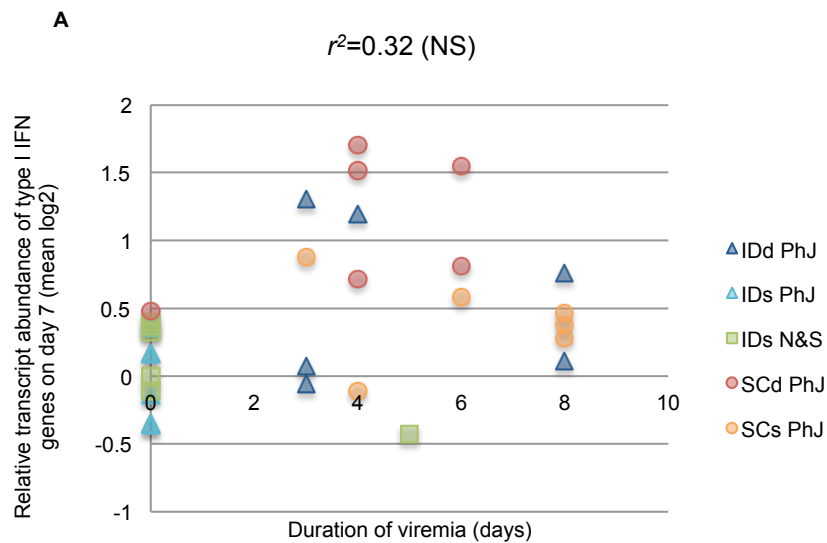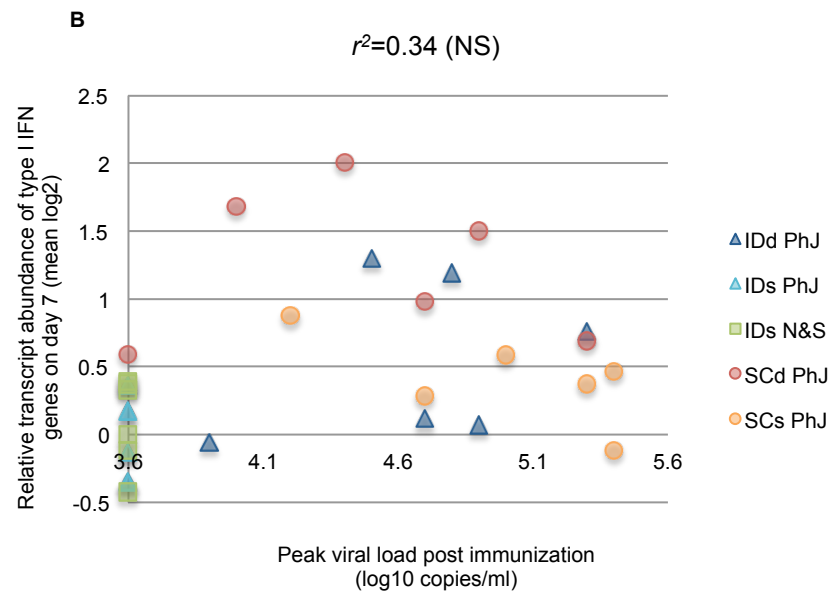

Supplement: S4 Fig — (PDF) [file pntd.0004731.s012.pdf]
